# Metastatic Voyage of Ovarian Cancer Cells in Ascites with the Assistance of Various Cellular Components

**DOI:** 10.3390/ijms23084383

**Published:** 2022-04-15

**Authors:** Kaname Uno, Shohei Iyoshi, Masato Yoshihara, Kazuhisa Kitami, Kazumasa Mogi, Hiroki Fujimoto, Mai Sugiyama, Yoshihiro Koya, Yoshihiko Yamakita, Akihiro Nawa, Tomohiro Kanayama, Hiroyuki Tomita, Atsushi Enomoto, Hiroaki Kajiyama

**Affiliations:** 1 Department of Obstetrics and Gynecology, Graduate School of Medicine, Nagoya University, Nagoya 466-8560, Japan; uno.kaname@med.nagoya-u.ac.jp (K.U.); iyoshi.shohei@med.nagoya-u.ac.jp (S.I.); kitami.kazuhisa@med.nagoya-u.ac.jp (K.K.); mogi.kazumasa@med.nagoya-u.ac.jp (K.M.); fujimoto.hiroki@med.nagoya-u.ac.jp (H.F.); yoshihiko-yamakita@kishokai.or.jp (Y.Y.); kajiyama@med.nagoya-u.ac.jp (H.K.); 2 Division of Clinical Genetics, Department of Laboratory Medicine, Lund University, 223-62 Lund, Sweden; 3 Spemann Graduate School of Biology and Medicine, University of Freiburg, 79104 Freiburg, Germany; 4 Discipline of Obstetrics and Gynecology, Adelaide Medical School, Robinson Research Institute, University of Adelaide, Adelaide 5005, Australia; 5 Bell Research Center, Department of Obstetrics and Gynecology Collaborative Research, Graduate School of Medicine, Nagoya University, Nagoya 466-8560, Japan; mai-sugiyama@kishokai.or.jp (M.S.); koya.yoshihiro@c.mbox.nagoya-u.ac.jp (Y.K.); nawa2005@med.nagoya-u.ac.jp (A.N.); 6 Department of Tumor Pathology, Graduate School of Medicine, Gifu University, Gifu 501-1194, Japan; kanayama.10mo16@gmail.com (T.K.); h_tomita@gifu-u.ac.jp (H.T.); 7 Department of Pathology, Graduate School of Medicine, Nagoya University, Nagoya 466-8560, Japan; enomoto@iar.nagoya-u.ac.jp

**Keywords:** ovarian cancer, ascites, spheroid, hetero-cellular spheroid, metastasis, anoikis, resistance to chemotherapy, mesothelial cell, macrophage, genetic evolution

## Abstract

Epithelial ovarian cancer (EOC) is the most lethal gynecologic malignancy and has a unique metastatic route using ascites, known as the transcoelomic root. However, studies on ascites and contained cellular components have not yet been sufficiently clarified. In this review, we focus on the significance of accumulating ascites, contained EOC cells in the form of spheroids, and interaction with non-malignant host cells. To become resistant against anoikis, EOC cells form spheroids in ascites, where epithelial-to-mesenchymal transition stimulated by transforming growth factor-β can be a key pathway. As spheroids form, EOC cells are also gaining the ability to attach and invade the peritoneum to induce intraperitoneal metastasis, as well as resistance to conventional chemotherapy. Recently, accumulating evidence suggests that EOC spheroids in ascites are composed of not only cancer cells, but also non-malignant cells existing with higher abundance than EOC cells in ascites, including macrophages, mesothelial cells, and lymphocytes. Moreover, hetero-cellular spheroids are demonstrated to form more aggregated spheroids and have higher adhesion ability for the mesothelial layer. To improve the poor prognosis, we need to elucidate the mechanisms of spheroid formation and interactions with non-malignant cells in ascites that are a unique tumor microenvironment for EOC.

## 1. Unique Characteristics Related to Poor Prognosis of Ovarian Cancer

Epithelial ovarian cancer (EOC) is the most lethal gynecologic malignancy with the highest case-to-fatality ratio [1,2]. More than 125,000 women die due to EOC each year worldwide, and this number has been predicted to rise to >250,000 by the year 2035 [3,4]. Although debulking surgery and repetitive chemotherapy are the standard treatments for EOC, the prognosis has not improved in the last decade, and only 20–30% of patients with the advanced disease live for over 5 years [4,5]. One reason for the poor prognosis must stem from several unique characteristics compared to other malignancies.

One of the most unique characteristics of EOC is the intraperitoneal fluid accumulation of ascites filled with EOC cells. EOC utilizes this fluid as a root to spread within the peritoneal cavity and create numerous intraperitoneal metastases, known as “transcoelomic dissemination [6,7]”, while metastasis beyond the peritoneal cavity is relatively rare [1]. Practically, transcoelomic dissemination using ascites is different from the hematogenous and lymphatic metastases found in other malignancies [8,9]. As accumulation of ascites is a common feature of EOC, the environment of the intraperitoneal cavity with ascites is key to understanding the unique characteristics.

The second unique point is rapid growth and early metastasis, finally leading to aggressive disease course. It is clinically hard to detect EOC at an early stage [10]. Gynecologists sometimes encounter patients with the advanced disease with peritoneal metastasis and ascites, although they had no signs or symptoms a few months ago. To date, a variety of clinical trials have challenged to diagnose EOC at the early stage, which include regularly checking for carcinoma antigen-125 (CA-125), a common tumor antigen of EOC, or surveying ovaries by transvaginal ultrasonography [3]. However, these trials could not ultimately reveal mortality reduction [11,12]. Unlike other common malignancies, including breast, colon, and gastric cancer, there is no reliable screening for detecting EOC [13], and this would cause the accumulation of ascites, finally allowing for transcoelomic dissemination.

Thirdly, the high recurrence rate and resistance to conventional chemotherapy are also the important feature of EOC, leading to poor prognosis [14]. Although tumor burden and progression speed is high, most EOCs are sensitive to the initial chemotherapy, and nearly 70% of patients can achieve complete remission after debulking surgery with repetitive chemotherapy [15,16]. However, over 80% of them develop a recurrent tumor within 3 years, which is an extremely high rate compared to that of breast cancer, 19% for example [1]. This high recurrence rate may, at least partially, be attributed to the stealth metastasis of EOC cells through ascites. More than 60% of the recurrence sites are still in the peritoneal cavity [8]. When recurrence occurs, EOC cells become resistant to chemotherapy, and re-accumulated ascites are known as one of the causes of this acquired chemoresistance [17]. Therefore, ascites and contained EOC cells possess a fundamental importance in progression and recurrence of EOC and should be recognized as a future research target.

Although EOC cells often draw attention solely, ascites are known to contain many non-malignant host cells, including macrophages, mesothelial cells, and lymphocytes [7,18,19,20], and the number of these cells is much higher than that of EOC cells [18,21]. Recently, the literature has shown that EOC cells form aggregated spheroids with these non-malignant cells [5,22,23], which is gaining significance in understanding the pathophysiology of EOC and creating new treatment approaches; however, these findings have not yet been summarized tidily.

In this review, we mainly discuss why EOC cells in ascites are related to poor prognosis from the viewpoint of their shape and function. The pathophysiology of ascites accumulation and various cellular components in ascites will also be summarized.

## 2. The Significance of EOC Cells in Ascites

When gynecologists suspect EOC, ascites cytology is usually performed during surgery. Positive ascites cytology of stage I EOC is diagnosed as stage IC3 because the presence of EOC cells in ascites is related to worse prognosis. Beyond staging, positive ascites cytology at the time of interval debulking surgery after chemotherapy is reported to have an independent negative prognostic impact [24]. Moreover, we have reported that even in stage II and III patients, positive ascites cytology during surgery was associated with progression and worse overall survival [25]. Conversely, the literature has shown that the presence of EOC cells in ascites is related to chemoresistance and cancer stemness [6,26,27]. Several authors have also reported that the amount of ascites was also related to poor prognosis and resistance to chemotherapies [28,29]. Therefore, the existence of EOC cells in ascites, which means positive ascites cytology, is important information in clinical settings, not only in the early stage but also in the advanced stage or after chemotherapy treatment.

## 3. Existing Form of EOC Cells in Ascites: Spheroids

**Figure 1:**
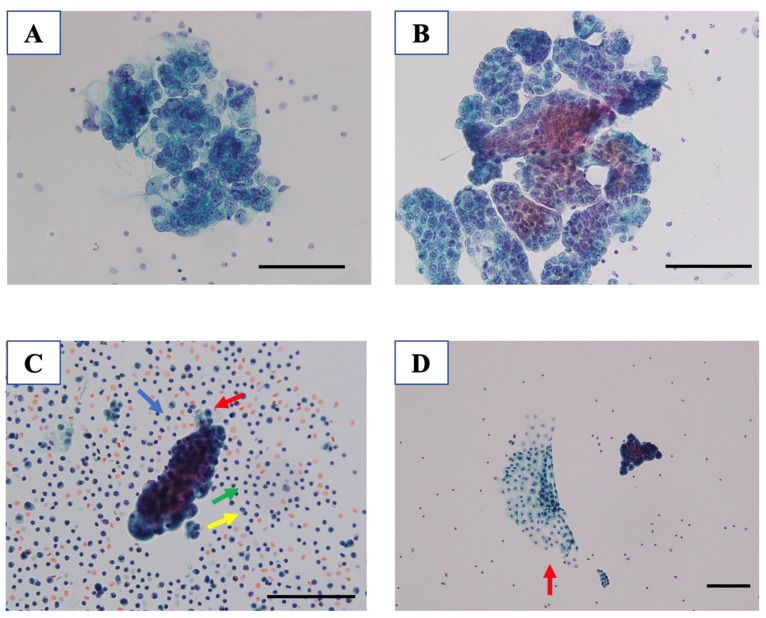
Representative images of cytology of malignant ascites of ovarian cancer and aggregated spheroids. Morphology is the most important information to distinguish malignant cells from non-malignant cells. Even in malignant ascites, there are many non-malignant cells, and some of them change their morphology by a variety of stimulations. (**A**,**B**) Papanicolaou staining of malignant ascites of epithelial ovarian cancer. Various sizes and shapes of spheroids are observed. (**C**) In malignant ascites, non-malignant cells, including macrophages (blue), mesothelial cells (red), lymphocytes (green), and neutrophils (yellow) are also detected. (**D**) Aggregation of mesothelial cells (red) is also observed. Scale bar: 100 μm. These data were acquired in our pathological unit using Papanicolaou staining from three different patients with advanced EOC (histological type were all high-grade serous ovarian cancer).

When EOC cells in ascites are observed with Papanicolaou staining, almost all tumor cells existed in the form of aggregated spheroids (Figure 1A), as suggested by previous studies and clinical practice [30,31,32]. The shape of these spheroids varied in size, circularity, and concentration in each patient. Although some studies reported that metastatic EOC cells originating from the primary tumor site exist as single cells [30,33,34,35,36], it is hard to find a so-called “single EOC cell” in ascites (Figure 1B), and few studies have revealed and discussed this fundamental point. Even gynecologists tend to suppose that EOC cells exist as single cells in ascites, and this misunderstanding may be a major problem in EOC research. For example, in most illustrations in previous studies, EOC cells in ascites have been schematically described as a single cell [35,36]. Some studies have warned that characteristics of EOC assessed in conventional 2D cultures are different from those in the human body or 3D spheroid cultures [37,38,39]. In addition, it is well known that some drug candidates evaluated in 2D culture do not show estimated potency when used in living organisms [27]. Moreover, to check the adhesion abilities of EOC cells, most experiments were performed using single cells [40,41]. In addition, upon close observation of ascites cytology, many non-malignant cells were observed, some of which also formed spheroids (Figure 1C,D). Specialists of cytology can distinguish these cells by morphology using Papanicolaou staining. For these reasons, we need to reconsider in vitro experimental protocols when assessing the model of EOC.

## 4. The Mechanisms Underlying Spheroid Formation in Ascites

Several studies have attempted to elucidate the mechanisms of spheroid formation of EOC. Epithelial-to-mesenchymal transition (EMT) induced by transforming growth factor (TGF)-β is reported as a key pathway [8,42,43,44,45,46]. When EOC cells detach from the ovary-formed spheroids, EOC cells decrease the expression of the epithelial marker, E-cadherin, and increase the expression of vimentin and N-cadherin [47]. Fluidic force of ascites is demonstrated to induce spheroid formation by decreasing the expression of E-cadherin and increasing vimentin expression [48]. Cao et al. have shown that TGF-β-stimulated EOC cells aggregate as spheroids quickly by tissue transglutaminase (TG2) [49]. TGF-β1/SMADs and NF-κB or PI3K pathways are also known to be activated in EOC spheroids [50,51]. Conversely, Kantak et al. have shown that E-cadherin is required for multicellular aggregation in squamous cell carcinoma [52]. Moffitt et al. recently have reported that detached EOC cells have a unique expression profile in which both epithelial and mesenchymal markers are co-expressed, including ZEB1, Twist, Slug, Snail, N-cadherin, and vimentin [32,42,53]. At the same time, several studies have revealed that cancer stem cells (CSCs) that express CD133 and aldehyde dehydrogenase (ALDH) have a high ability to form spheroids [54,55]. The relationship between spheroids and CSCs will be discussed separately later in this review.

In contrast, Habyan et al. have suggested that multicellular spheroids arise from collective detachment, rather than aggregation in the abdominal cavity [47]. Moreover, some authors have revealed that cancer-associated fibroblasts (CAFs) or macrophages drive EOC spheroids in ascites [5,22]. They showed that these non-malignant cells become the core component of the spheroids. These different mechanisms can result in various sizes and shapes of spheroids in EOC cells, as shown in Figure 1. However, we have not fully understood whether these spheroids detach from the primary or metastatic sites, and how long these spheroids can survive in the presence of ascites even under chemotherapy treatment. Further research is needed to reveal the mechanisms of spheroid formation of EOC in ascites.

Several studies have tried to reveal the genetic evolution to form malignant spheroids in ascites and to spread to the peritoneal cavity. *TP53* mutation, which is the most frequently mutated tumor suppressor gene in human cancer, is believed to be the earliest tumorigenic driver event in EOC, and up to 95% of tumors are known to have somatic *TP53* mutation [56]. Moreover, the high rate of *TP53* mutation and *BRCA* deficiency has been documented to lead to genomic instability, highly individual evolutionary trajectories, and extensive intratumoral heterogeneity [57,58]. The loss of regulator components of the homologous recombination DNA-damage repair pathway, including *BRCA1/2*, is also a well-known factor for tumor development [59]. The importance of the mutation of *TP53*, *BRCA1/2*, and *PTEN* in peritoneal dissemination has been shown as a genetic factor by several mice models [60,61]. Additionally, the loss of *PTEN* induces spheroid formation in fallopian epithelial cells [62], contributing to anoikis resistance. These mutations in ovarian cancer suppressor genes can cause further mutations of various oncogenes or other suppressor genes, which are reported to be associated with therapy resistance [63]. Therefore, EOC exhibits highly diverse genomic heterogeneity even prior to treatment [57,58]. Conversely, several studies tried to reveal the genetic evolution from primary tumors to peritoneal dissemination using next-generation sequencing. Some studies have shown that the mutational characteristics revealed by whole-exome sequencing or whole-genome sequencing are similar between primary tumors and matched disseminated tumors [64,65]. Furthermore, other studies analyze the heterogeneity of matched primary, peritoneal disseminated lesions, and ascites cells; the genetic pattern of spheroids-forming EOC cells has been shown to be extremely similar when compared to primary and disseminated lesions [66,67]. These data indicate that little genetic alternation accumulate from tumor cells in primary lesions to those in spheroids in ascites and disseminated sites, showing the uniqueness of transcoelomic metastasis. Meanwhile, through genetic analyses with matched spheroids in ascites and solid tumors (primary and peritoneal dissemination), the tumor suppressor gene *FAT3*, coding an atypical cadherin, is found to be upregulated only in disseminated lesions. In addition, a specific subset of mesothelial genes, including calretinin (*CALB2*) and podoplanin (*PDPN*), are expressed in spheroids from ascites and disseminated lesions [66]. Thus, studies regarding EOC spheroid formation are accumulating; however, further studies are still necessary to elucidate the mechanisms of spheroid formation and peritoneal dissemination, including genetic and epigenetic changes among primary spheroids in ascites and peritoneal disseminated lesions.

## 5. Advantages of EOC Spheroid Formation in Ascites

Why do almost all EOC cells need to form spheroids in ascites? In this section, the answers to this fundamental question are summarized. There are several mechanisms that benefit from forming spheroids rather than remaining as single cells in ascites.

### 5.1. EOC Cells Forming Spheroids Are More Likely to Survive in Ascites

The most important and lethal reason for spheroid formation is thought to be anoikis resistance. For epithelial cells, interactions between the extracellular matrix (ECM) and anchor proteins provide essential signals that promote survival and growth [32,52,68]. The loss of cell–ECM interaction is a strong inducer of apoptosis, and it is independent of the p53 pathway. Anoikis is reported to be caused by a mitochondrial-activated pathway, which leads to DNA fragmentation [52,69]. In ascites, EOC cells need to be aggregated in order to avoid anoikis [1,52]. Therefore, adherent cells spontaneously aggregate in a manner consistent with a normal/natural survival response [6,68]. Thus, spheroids are more resistant to anoikis and have a survival advantage over single cells in ascites [47,52]. EOC cells grown as spheroids survived over 10 days, whereas single cells failed to grow beyond 2 days in an anchorage-independent condition [70]. Some authors have shown that most single EOC cells die after intraperitoneal injection, and the ability to induce peritoneal metastasis depends on anoikis resistance [5,68,71]. The authors also showed that EOC cells were almost always detected as forms of spheroids as well.

Mechanistically, knockdown of STAT-3 or CDCP1 affects spheroid formation and results in a significant reduction in the number of surviving cells in 3D culture [72,73]. Kim et al. showed that rapid increases in superoxide dismutase2 (SOD2) under regulation by SIRT3 prevented mitochondrial superoxide surges in detached cells, sustained anchorage-independent growth, and colonization of the peritoneal cavity [69]. The TGF-β pathway is also reported to affect resistance to apoptosis through EMT [74]. Through spheroid formation, EOC cells seem to acquire resistance against anchorage-dependent cell death, survive and grow in ascites, and metastasize to the peritoneal cavity.

### 5.2. Increased Ability to Adhere and Invade the Mesothelial Layer

Adhesion and invasion into the mesothelial layer is an essential step for peritoneal metastasis of EOC cells in ascites [1]. The peritoneal cavity is surrounded by a single layer of mesothelial cells, which acts as an initial barrier for cancer cells or outsiders [75]. Spheroids are thought to be critical in the sequential steps of developing peritoneal metastasis of EOC [49,76]. Some studies have revealed that spheroids can more easily adhere to the peritoneal cavity compared to single cells [70,77], and the invasion into the monolayer of mesothelial cells is also promoted [31,76].

Integrin signaling has been shown to relate not only to spheroid formation, but also to disaggregation [31,78]. Some authors have revealed that spheroids in ascites adhere to the mesothelial layer via the β1 integrin subunit [78]. Expression of α2β1 integrin influenced spheroid disaggregation and activated MMP2 and MMP9 to invade the submesothelial layer [31,70]. TGF-β, which is present in ascites, stimulates EOC cells and promotes invasiveness through EMT [45]. Moreover, mesenchymal N-cadherin-expressing spheroids are reported to efficiently rupture peritoneal mesothelial cells [76,79]. These mesenchymal phenotypic EOC cells, called “leader cells”, may take advantage of invading the mesothelial layer with actin-rich invadopodia and other EOC cells following as “follower cells” [32]. In addition, spheroids of EOC cells are known to develop ICAM-1 to interact with mesothelial cells in the peritoneal cavity [5,50]. However, the mechanisms by which spheroids invade the mesothelial layer are not fully understood. Further studies are required to clarify these mechanisms and to develop a future therapeutic target. Moreover, most disseminated EOC cells are well known to invade adipose-rich tissues, such as omentum. Recently, studies showing the importance of the interaction between EOC cells and adipocytes in EOC progression, invasion, and metastasis have accumulated [9,80,81]. However, studies using only single EOC cell models in evaluations and studies revealing the interaction between EOC spheroids and adipocytes are lacking.

### 5.3. Resistance for Chemotherapy Related to Stem Cell Ability

Many studies have reported that 3D-cultured spheroids exhibited greater resistance to chemotherapies [6,26,27,37,82,83,84,85]. When grown in 3D culture, cancer cells can acquire an additional resistance to apoptosis, which is thought to mimic the chemoresistance observed in solid tumors [39,86,87]. Previous studies have revealed that spheroid formation promotes chemoresistance because EOC cells in spheroids have the stem cell-like features when compared to those in 2D culture [31,47,55,88,89]. Although no universal stem cell markers were found, ALDH1, CD44, CD117, CD133, and Nanog are thought to be the candidates in EOC [26,90,91]. Some studies have shown that a slow cell cycle in EOC cells in spheroids is related to chemoresistance [55,92]. EOC cells in detached condition had fewer cells in the G2/M and S phases, which represents a slow cell cycle and/or quiescent proliferation compared with those in the adherent condition [6,37,69,93]. Spheroids are also known to have high drug efflux systems, including MDR1 [26]. Mechanistically, EOC spheroids were shown to be in proliferative arrest but invasive, and Bcl-2 [92] or pyruvate dehydrogenase kinase 4 (PDK4) [55] was detected to be the key molecule in chemoresistance, stemness, and promoting metastasis. Dissociated cells of EOC spheroids after chemotherapy had a high rate of stem cell markers and resistance to chemotherapy [54,90,94]. Therefore, some researchers suggested the possibility that these EOC spheroids may contribute to developing tumor recurrence after treatment [1,6,84,95]. Conversely, other studies revealed that the chemoresistance of spheroids is caused by slow penetration of anti-cancer drugs into the 200 µm of spheroid layers, resulting in low concentrations of active agents in the vicinity of tumor cells [83,89]. Since chemotherapeutic agents do not address anchorage- or vascular-independent growth conditions [6], EOC spheroid formation is considered beneficial for obtaining stem cell properties and resistance to chemotherapy.

## 6. Ascites: Why Does It Increase and What Are Its Constituents?

Ascites is the most important and fundamental characteristic of EOC, and most patients with advanced EOC present with massive ascites [15,28]. Ascites contains not only EOC cells but also numerous non-malignant cells and acellular components [35,36]. Recently, increasing attention has been given to ascites and its role in the progression of EOC. In this section, we discuss the mechanisms of ascites accumulation, as well as cellular and acellular components, which are thought to promote tumor proliferation, anti-apoptosis, adhesion, invasion, and chemoresistance.

### 6.1. The Mechanisms of Ascites Accumulation

Even in healthy women, a small amount of fluid exists in the peritoneal cavity, which is the space between the parietal and visceral layers [43]. Ascites is thought to be important to keep the condition of the intraperitoneal cavity stable [96,97]. The amount of ascites is controlled by extraction from capillaries and absorption to the lymphatic system through the mesothelial layer [43]. Thus, ascites accumulation occurs when this balance is broken. Hepatic cirrhosis and invasion of some types of malignancies into the peritoneal cavity are well known to develop ascites [98]. Among these malignancies, EOC is the most common cause of ascites compared to pancreatic, colorectal, liver, and endometrial cancers [33,35]. Although hepatic cirrhosis induces high blood pressure of capillaries and low protein levels in blood vessels, pathogenesis of malignant ascites is more complexed [96].

In malignant ascites, various kinds of cytokines, including TGF-β, are also increased, and these cytokines cause inflammation [99]. Mesothelial cells lining the peritoneal cavity are also thought to play an important role in controlling the amount of ascites [97,100], and inflammation of mesothelial cells disrupt their drainage function, resulting in the accumulation of ascites [43]. Increased capillary permeability by the upregulation of vascular endothelial growth factor (VEGF) also increases the amount of ascites [2]. Anti-VEGF drugs can be used to control ascites in clinical settings [34]. Clinically, increased ascites causes severe symptoms in patients, and nearly 50% of deaths in EOC patients are related to cachexia with the massive accumulation of ascites [28,29]. Therefore, understanding the mechanism and developing new treatments for ascites accumulation are strongly demanded.

### 6.2. Cellular Components in Ascites

In malignant ascites, many cellular components are associated with the condition of the peritoneal cavity as an ecosystem of tumor microenvironment [40,101,102], and these cellular components in ascites are different from those in other parts of the human body [7,34,103]. The intraperitoneal cavity is covered by a single layer of mesothelial cells that line behind a connective tissue, consisting of adipocytes, fibroblasts, endothelial cells, and immune cells [101,102,103,104,105]. Among them, mesothelial cells and macrophages are reported to be key components of malignant ascites [7,18,19,20]. The cellular components of malignant ascites were counted and reported as follows: 37% lymphocytes, 29% mesothelial cells, 32% macrophages, and few neutrophils and EOC cells [21]; fibroblasts and adipocytes were not described in the literature. Although the percentage is different for each patient, this study is important in revealing the rate of cellular components, and there exist more non-malignant cells than cancer cells [18,21]. When we see cytological slides of ascites, we can detect not only EOC cells as spheroids but also lymphocytes, mesothelial cells, and macrophages (Figure 1C,D). Most of these cells can be distinguished by morphology using Papanicolaou staining in clinical settings, although some of them, especially reactive mesothelial cells, are difficult to distinguish from malignant cells [106]. Mesothelial cells can show reactive change due to a variety of stimulations. Several immunohistochemical stainings are used to distinguish reactive mesothelial cells from malignant EOC or mesothelioma cells because reactive mesothelial cells show a marked enlarged nucleus and hyperchromesia, which are similar to malignant cells [106,107]. The number of lymphocytes and neutrophils varies because these numbers are affected by blood inclusion.

Some researchers might believe that the cellular components in ascites are similar to those in solid tissues. In a previous review of ascites, fibroblasts, endothelial cells, adipocytes, and mesenchymal cells were illustrated as floating cells in ascites [33,34,35,36]. However, in view of pathology, fibroblasts, adipocytes, and endothelial cells are not expected to exist in ascites. Although several studies have attempted to show the existence of fibroblasts in ascites through positive markers of αSMA [22], this marker is not specific to fibroblasts, and the origin of “so-called” cancer-associated fibroblasts (CAFs) can be varied, i.e., originating not only from fibroblasts [108]. As suggested above, the tumor microenvironment in ascites is different from that of other malignancies. For example, the physiological functions of mesothelial cells, which are the main cellular components of ascites, are diverse [97,109,110,111]. In pathological units, malignant cells, activated mesothelial cells, and macrophages are often difficult to distinguish from each other because the morphology of mesothelial cells can change easily depending on the peritoneal conditions [100,112,113]. In the presence of TGF-β, mesothelial cells increase in size, become permeable, and change into spindle-shaped CAFs-like cells due to their mesenchymal change [19,30,105,114]. Some studies have revealed that free-floating mesothelial cells attached to the injured peritoneum and repopulated [97,109,109]. Other studies have revealed that floating mesothelial cells in malignant ascites experience mesenchymal transformation and express both mesenchymal markers, αSMA and calretinin. They concluded that activated mesothelial cells are one of the subtypes of CAFs in the metastatic region [114,115,116]. Regarding the unique function of mesothelial cells concerning the engulfment of dying cells, *Staphylococcus aureus* and asbestos fibers are also reported [111,117], similar to macrophages in the peritoneal cavity. Consequently, we must recognize that the peritoneal cavity and ascites are quite different from those of other parts of the human body [118].

Microenvironments in the peritoneal cavity are suitable “soil” for EOC cells. Some authors reported that one of the reasons that mortality of EOC has not significantly improved during the last decade is attributed to poor understanding of interactions between EOC cells and the unique surrounding environment [19,32]. As EOC cells derived from the primary site already interact with non-malignant cells in the ascites before metastasis to the peritoneal wall [103], they should be dramatically affected by these surrounding cells. There are few studies on this topic thus far; therefore, we need further clarification of these interactions.

### 6.3. Acellular Components in Ascites

Acellular components are also present in ascites. Ascites is complex and is mainly derived from heterogeneous fluids that contain a variety of cytokines, chemokines, growth factors, and other soluble factors, such as lysophosphatidic acid (LPA) [119,120]. Various cytokines, including VEGF, IL-6, IL-8, IL-10, and TGF-β, are also secreted from EOC cells and non-malignant cells. Additionally, cell-free DNA and ECM-related components are reported to exist at high concentrations and support EOC cells for adhesion and metastasis [36,40,121]. Recently, as one of the acellular components in EOC ascites, extracellular vesicles (EV), or the exosome, has received a lot of attention. It is well known that cancer cells secrete more EV than non-malignant cells. EV contains a variety of proteins, lipids, microRNAs, microDNAs and transcriptional factors [122]. These EVs provide a suitable environment for tumor development and disseminations. Several studies showed that the contents of EVs from EOC cells could change the microenvironment of the abdominal cavity, including alterations of the macrophage phenotype [123] and destruction of the mesothelial barrier [124] to promote abdominal disseminations. Although the importance of EVs in ascites for diagnosis or treatment is studied intensively, the effect on tumor development has not been fully elucidated [122]. These cellular and acellular components make a unique tumor microenvironment in ascites, which remarkably distinguishes EOC from other malignancies.

## 7. Various Cellular Components of Spheroid in Ascites

As described above, there are a variety of cellular components in ascites, including mesothelial cells, macrophages, and lymphocytes. Recently, some reports suggest that cellular components of EOC spheroids are not only EOC cells but also non-malignant cells, especially in the core of these spheroids. Several studies have shown that there are αSMA-positive “fibroblasts” from primary EOC spheroids. Gao et al. showed that these fibroblast-like cells were also positive for fibroblast activation protein (FAP) in dual immunohistochemistry [22]. Han et al. showed αSMA-positive cells in immunohistochemical analysis of paraffin-embedded primary samples [125]. The authors of these reports suggested that αSMA or FAP were specific to fibroblast cells, even in ascites. However, there are also αSMA- or FAP-positive cells other than fibroblasts, including EOC itself and activated mesothelial cells [23,126]. When the mesothelial layer is damaged due to operational procedures or internal inflammation, mesothelial cells become activated and recover the damaged site. These activated mesothelial cells become positive for αSMA by mesenchymal transformation. Likewise, floating mesothelial cells in ascites are also reported to be positive for αSMA [30,109,127]. Therefore, mesothelial cells can interact with EOC cells in ascites, and attention should be paid concerning cellular origin of fibroblast-like cells in EOC spheroids. To the best of our knowledge, only two studies have suggested the presence of mesothelial cells in EOC spheroids in ascites [23,128]. However, these studies were limited to reveal the existence of mesothelial cells in the spheroids because they only defined mesothelial cells from a small part of positive staining for calretinin or αSMA. Although calretinin is thought to be a specific marker of mesothelial cells [20,95], some studies revealed that EOC cells were also positive for calretinin, and this marker is not highly sensitive for detecting mesothelial cells [129,130]. Dividing mesothelial cells, especially in activated conditions from EOC cells, is usually difficult because these cells share cellular origins [34,101]. Therefore, some EOC cells become positive for mesothelial markers and vice versa. A variety of markers are usually used to distinguish them in clinical pathology, including EpiCAM, cytokeratin-5/6 or 8, vimentin, PAX-8, podoplanin, and HBME-1 [95,101]. These various stainings are necessary for detecting mesothelial cells from EOC spheroids in experimental settings.

Macrophages are also major cellular components of malignant ascites. Yin et al. and Raghavan et al. reported that EOC spheroids in ascites contain macrophages. They showed macrophages in EOC spheroids by the Cre-mouse model or staining CD68 for primary samples. They revealed that these macrophages exist in the core of the spheroids and EOC cells surround the macrophage core. They also showed that macrophages in ascites did not exist in the first two weeks after injection of EOC cells, and the type of macrophages changed from M1 to M2 macrophages during spheroid formation [5,91].

Several studies have demonstrated that cancer cells can form more aggregated and compact spheroids in 3D culture when they grow with various non-malignant cells, including macrophages, fibroblasts, and mesothelial cells [44,131,132,133,134]. Above all, these “hetero-cellular” EOC spheroids showed higher adhesion ability for the mesothelial layer [5,22] and resistance to chemotherapy [91] through direct and indirect interactions in ascites before intraperitoneal metastasis [7]. Interactions with the microenvironment have been shown to play a significant role in determining the fate of EOC cells that leaves the primary tumor site and metastasizes to a distant site [23].

## 8. Potential Therapeutic Targets and Future Perspective

In ascites, there are various cellular and acellular components that create unique tumor microenvironments of EOC. EOC cells detached from the primary site interact with these components before developing peritoneal metastasis. As most EOC cells exist as spheroids in the ascites fluid, the unique characteristics of spheroids described above can be directly related to poor prognosis. Conversely, these characteristics of EOC spheroids can also be applied to a novel treatment approach that is different from that for other tumors. For example, the blockage of Wnt signaling or attenuation of STAT-3 can lead to disaggregation of spheroids and increase sensitivity to chemotherapy [72]. Other studies showed a possibility that EOC cells become sensitive to chemotherapy when breaking the spheroids [93,95]. As EMT causes spheroid formation, many researchers have been focusing on this pathway, including the PI3K/Akt and TGF- β signaling pathways in the context of heterogenous cell-to-cell crosstalk [114,135]. Recently, Kitami K, et al. have shown that vitamin D can reverse the EMT condition of mesothelial cells through the interaction of EOC cells [136]. Although the effect for spheroid has not been demonstrated, these approaches combined with conventional chemotherapy may condition the whole peritoneal environment and reduce the ability to form EOC spheroids, including ascites, leading to the control of disease progression [73]. Moreover, as extraperitoneal metastasis rarely occurs, unique treatment approaches focusing on the intraperitoneal cavity have been conducted thus far to tackle EOC peritoneal disseminations. Some randomized studies were conducted to reveal the efficacy of intraperitoneal treatment. The Gynecologic Oncology Group (GOG) 172 study showed the improved survival rate with intraperitoneal administration in 2006 [137], although GOG252 did not reveal superiority compared to the group with intravenous administration [138]. Heated anti-cancer agent injections to the peritoneal cavity after operation is another example, because some studies showed the pharmacokinetics and effectiveness of intraperitoneal chemotherapy [139,140]. Hyperthermic intraperitoneal chemotherapy (HIPEC) is the standard treatment for pseudomyxoma peritonei [141]. The effectiveness of HIPEC has also been reported in EOC [139,140]. In addition to treating peritoneal tumors, this direct approach can be effective in suppressing EOC spheroids in ascites versus standard intravenous chemotherapy. This is because most EOC spheroid sizes are up to 300 μm, and these intraperitoneal anti-tumor agents could penetrate at a depth of 5 mm [142]. Further research is needed to target EOC spheroids in ascites, including hetero-cellular spheroids.

**Figure 2:**
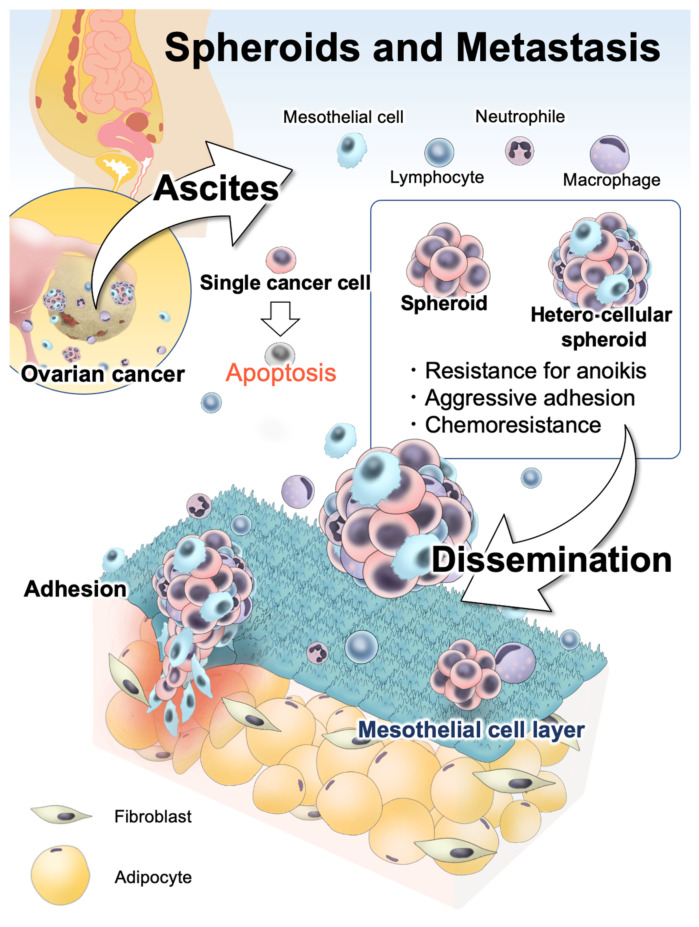
Image of EOC cells in ascites detached from primary cite. The microenvironment in ascites is complex and dynamic. EOC cells form spheroids for surviving anchorage-independent conditions, the ability to adhere to the mesothelial layer, and resistance to chemotherapy. In ascites, multiple types of non-malignant cells, including mesothelial cells, lymphocytes, neutrophils, and macrophages, support EOC metastasis. Some of EOC spheroids are composed of not only EOC cells but also non-malignant cells. These hetero-cellular spheroids are reported to be more aggressive in their abilities for adhesion and invasion.

We illustrated the current model of EOC cells and their forms in ascites (Figure 2). The interaction between EOC spheroids and non-malignant cells in ascites has not been investigated enough in detail. The microenvironment in ascites seems to offer complex and dynamic support for EOC cells, leading to the unique features of EOCs related to poor prognosis. To elucidate them and to achieve new treatment strategies, ascites components, especially EOC spheroids that interact with cellular and acellular components, can be a key to improve the prognosis of patients with EOC in the future.
